# Machine learning characterization of a rare neurologic disease via electronic health records: a proof-of-principle study on stiff person syndrome

**DOI:** 10.1186/s12883-024-03760-7

**Published:** 2024-08-03

**Authors:** Soo Hwan Park, Seo Ho Song, Frederick Burton, Cybèle Arsan, Barbara Jobst, Mary Feldman

**Affiliations:** 1 Geisel School of Medicine at Dartmouth, Hanover, NH USA; 2 Department of Neurology, Dartmouth Health, Lebanon, NH USA; 3 Department of Psychiatry, Beth Israel Deaconess Medical Center, Harvard Medical School, Boston, MA USA; 4 Department of Psychiatry, University of California Los Angeles Health, Los Angeles, CA USA; 5 Department of Psychiatry, Oakland Medical Center, Kaiser Permanente, Oakland, CA USA

**Keywords:** Rare neurologic disease, Stiff person syndrome, Machine learning, Electronic health records

## Abstract

**Background:**

Despite the frequent diagnostic delays of rare neurologic diseases (RND), it remains difficult to study RNDs and their comorbidities due to their rarity and hence the statistical underpowering. Affecting one to two in a million annually, stiff person syndrome (SPS) is an RND characterized by painful muscle spasms and rigidity. Leveraging underutilized electronic health records (EHR), this study showcased a machine-learning-based framework to identify clinical features that optimally characterize the diagnosis of SPS.

**Methods:**

A machine-learning-based feature selection approach was employed on 319 items from the past medical histories of 48 individuals (23 with a diagnosis of SPS and 25 controls) with elevated serum autoantibodies against glutamic-acid-decarboxylase-65 (anti-GAD65) in Dartmouth Health’s EHR to determine features with the highest discriminatory power. Each iteration of the algorithm implemented a Support Vector Machine (SVM) model, generating importance scores—SHapley Additive exPlanation (SHAP) values—for each feature and removing one with the least salient. Evaluation metrics were calculated through repeated stratified cross-validation.

**Results:**

Depression, hypothyroidism, GERD, and joint pain were the most characteristic features of SPS. Utilizing these features, the SVM model attained precision of 0.817 (95% CI 0.795–0.840), sensitivity of 0.766 (95% CI 0.743–0.790), F-score of 0.761 (95% CI 0.744–0.778), AUC of 0.808 (95% CI 0.791–0.825), and accuracy of 0.775 (95% CI 0.759–0.790).

**Conclusions:**

This framework discerned features that, with further research, may help fully characterize the pathologic mechanism of SPS: depression, hypothyroidism, and GERD may respectively represent comorbidities through common inflammatory, genetic, and dysautonomic links. This methodology could address diagnostic challenges in neurology by uncovering latent associations and generating hypotheses for RNDs.

## Background

Of more than 6,000 rare diseases that affect about 6% of the global population [1, 2], at least half are life-threatening or chronically debilitating dysfunctions of the central or peripheral nervous system [3]. However, due to the medical communities’ limited expertise in unusual disease presentations and insufficient diagnostic tools, rare neurologic diseases (RNDs) are frequently detected with a delay. Despite the socioeconomic burden of such diseases, the disorders’ rarity hinders the accrual of sufficient statistical power appropriate for systematic exploration of their pathological and clinical features, which are crucial for optimizing diagnostic accuracy and precision [4].

Recent advancements in artificial intelligence and machine learning (ML) have introduced novel strategies to study the pathologies and treatment outcomes for RNDs. Applying ML to electronic health records (EHR) is demonstrating increasing promise in the accurate detection of rare diseases and is gaining traction as decision support systems due to its strengths in extracting knowledge and identifying otherwise elusive associations [5–7]. Such capabilities are offering opportunities to mine clinical data, even retrospectively, to augment the diagnostic clarification and confirmation of rare diseases and validate co-occurring characteristics amongst affected individuals [8]. These insights bolster the case for ML’s increasingly larger role in decoding rare diseases and perhaps even inspiring fresh hypotheses based on newly unearthed correlations.

Stiff person syndrome (SPS) is an RND characterized by axial muscle stiffness and painful spasms, affecting only one to two in a million annually [9, 10]. While hallmark electromyography findings, symptomatic alleviation from benzodiazepine therapy, and the presence of autoantibodies against glutamic-acid-decarboxylase-65 (anti-GAD65) suggest SPS, diagnosis still relies on subjective clinical judgment, given the condition’s variable and poorly understood presentation [11, 12]. This ambiguity often leads to misdiagnosis and significant delays in diagnostic confirmation (mean delay of 6.2 years) and treatment initiation. Like many other RNDs, the rarity of SPS leads to insufficient statistical power, challenging the identification of relevant disease interrelationships. In this retrospective proof-of-principle study, we showcased the utility of ML models on EHR to overcome the constraints of conventional statistical tests and highlight the capability of such models to identify potential clinical features that may bear comorbid associations in the pathology of SPS.

## Methods

### Subject characteristics

To date, there exists no consensus on the ‘ideal’ sample size in ML analyses of EHR-based clinical data [13]. While higher sample sizes minimize type II errors, rare diseases are prone to mislabeling which can exacerbate type I errors, which are equally, if not more worrisome than insufficiencies in statistical power [14]. To that end, in this single-center retrospective study, we maintained a strict inclusion criterion with carefully selected patients with SPS and controls based on positive serum antibody titers to avoid any mislabeling of data and preserve model performance. Thus, our retrospective cohort consisted of 48 patients who were seen at Dartmouth Health from January, 2010 to January, 2020 and had elevated serum anti-GAD65 titers (≥ 0.03 nmol/L). Of these 48 patients, 23 were diagnosed with SPS by neurologists. All subjects’ past medical history (problem list) items from the EHR were binarized (1 if present; 0 otherwise), resulting in 319 unique items. This study was approved by the Committee for the Protection of Human Subjects at Dartmouth Health (STUDY02000166) and followed the Strengthening the Reporting of Observational Studies in Epidemiology (STROBE) reporting guideline.

### Data analysis and feature selection

Patient demographics as well as relevant binarized clinical characteristics based on previous literature on comorbidities of SPS (e.g., autoimmune processes) were evaluated via t-tests and Fisher’s exact tests in SPSS [15, 16].

To identify the key features that best characterize SPS among the 319 binarized items, we applied a contribution selection algorithm (CSA), an iterative process that optimizes a classifier’s performance by progressively evaluating and reranking features [17]. We adopted a backward elimination approach with a support vector machine (SVM) classification model featuring a Gaussian kernel for its superior versatility relative to linear or polynomial kernel functions [18].

Beginning with 319 features, each iteration of the CSA performs three tasks: 1) computes the SHapley Additive exPlanation (SHAP) values, which reflect the relative contribution of individual features, 2) assesses classifier performance, and 3) eliminates the feature with the lowest SHAP value. Derived from cooperative game theory, a feature’s SHAP value signifies the feature’s impact on the model’s prediction, reflecting the shifts in prediction in response to the presence or absence of that feature [19].

**Fig. 1:**
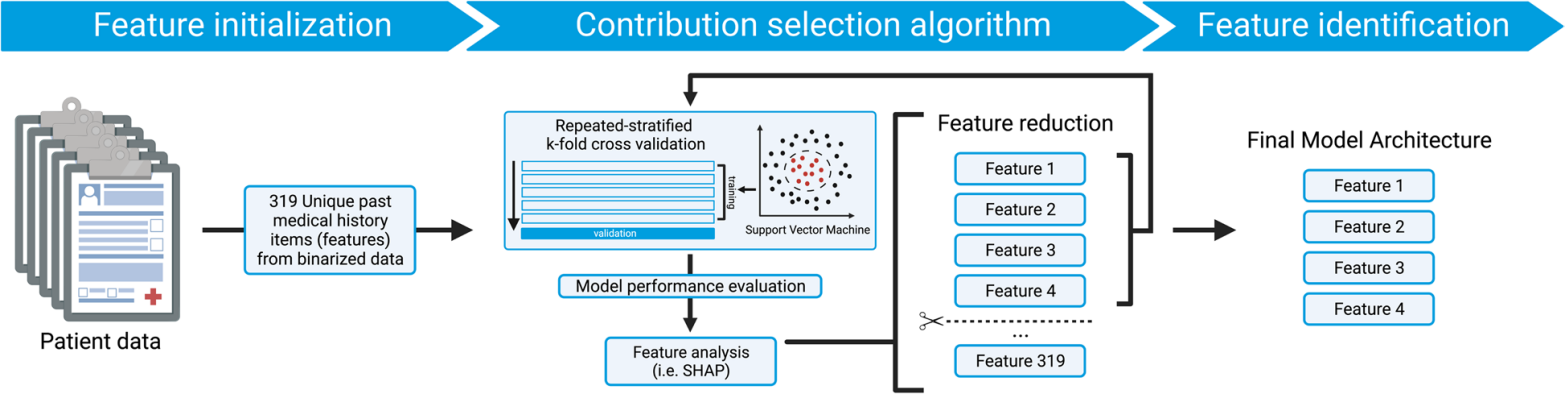
Architectural overview of support vector machine-based contribution selection algorithm (CSA) pipeline. After binarization of 319 medical history items from all subjects, each iteration of CSA computes the SHapley Additive exPlanation (SHAP) values for each feature, assesses the model’s performance in classifying patients with stiff person syndrome vs controls, and eliminates feature with the lowest SHAP value. Features in the best-performing model are identified

Using the “shap” package and custom scripts in Python3, we implemented our algorithm, iterating through 319 models of progressively fewer features [19]. We overcame our small sample size limitation by rigorously training our Gaussian kernel-based SVM model with both SPS and non-SPS patient cases and validating its predictions via a repeated-stratified fourfold validation scheme. Performance metrics and 95% confidence intervals for each model were calculated from 200 rounds of bootstrapping. The features employed by the highest-performing model were subsequently identified, and their respective impact on the models’ decision was visualized. The overall architecture for our feature identification pipeline is shown in Fig. 1.

## Results

### Subject characteristics

Baseline demographics and relevant clinical features of 23 patients with SPS and 25 anti-GAD65 controls were analyzed. No statistically significant differences were detected in terms of demographics, serum anti-GAD65 titer, or clinical/psychiatric characteristics/comorbidities (*p* > 0.05) (Table 1).

**Table 1:** Demographics and clinical characteristics

	**SPS + **(N = 23)	**SPS-** (N = 25)	***p*-value**
**Demographic Characteristics**			
** Age –** years, mean (SD)	54.3 (10.7)	45.9 (21.6)	0.090
** Female sex –** no./total no. (%)	13/23 (57)	12/25 (48)	0.578
** White ethnicity –** no./total no. (%)	18/23 (78)	18/25 (72)	0.743
**Clinical Characteristics (Known comorbidities)**			
** Anti-GAD65 titer (serum)** nM, mean (SD)	161.7 (521.1)	93.8 (299.1)	0.588
** Diabetes mellitus type 1** no./total no. (%)	1/23 (4)	2/25 (8)	1.000
** Graves disease** no./total no. (%)	1/23 (4)	1/25 (4)	0.180
** Hashimoto’s thyroiditis** no./total no. (%)	2/23 (9)	2/25 (8)	0.180
** Celiac disease** no./total no. (%)	0/23 (0)	2/25 (8)	0.180
** Epilepsy** no./total no. (%)	1/23 (4)	2/25 (8)	0.180
** Limbic encephalitis** no./total no. (%)	1/23 (4)	1/25 (4)	0.180
** Autoimmune encephalitis** no./total no. (%)	0/23 (0)	1/25 (4)	0.180
**Clinical Characteristics (Psychiatric)**			
** Trauma/stress spectrum** no./total no. (%)	4/23 (17)	2/25 (8)	0.407
** Anxiety spectrum** no./total no. (%)	9/23 (39)	7/25 (28)	0.543
** Schizophrenia spectrum** no./total no. (%)	4/23 (17)	1/25 (4)	0.180
** Unipolar mood spectrum** no./total no. (%)	12/23 (52)	7/25 (28)	0.140
** Bipolar mood spectrum** no./total no. (%)	2/23 (9)	0/25 (0)	0.224
** OCD spectrum** no./total no. (%)	2/23 (9)	0/25 (0)	0.224
** Personality disorders** no./total no. (%)	3/23 (13)	0/25 (0)	0.102
** Somatic symptom disorders** no./total no. (%)	3/23 (13)	0/25 (0)	0.102

Statistical differences were computed via Welch’s t-tests or Fisher’s exact tests

### Identification of relevant features

For each step of CSA, an SVM model was trained using the remaining features and validated using a stratified fourfold cross-validation scheme. Generally, filtering out non-discriminatory features from the models increased prediction metrics, as expected.

**Fig. 2:**
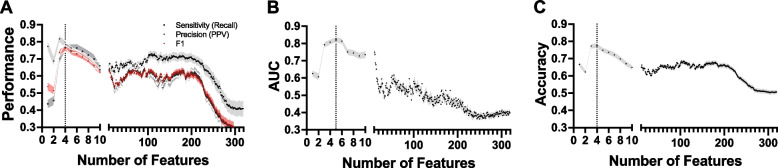
Determination of Ideal Feature Set Through Evaluation of Model Performance. Model performance was calculated at every iteration of the backward elimination feature selection process. **A** F1-score and sensitivity are optimized at four features with 0.761 (95% CI, 0.744 to 0.778) and 0.766 (95% CI, 0.743 to 0.790), respectively, while precision reached a peak with three features with 0.817 (95% CI, 0.795 to 0.840). **B** Area under the curve (AUC) is optimized at five features with 0.808 (95% CI, 0.791 to 0.825). **C** Accuracy is optimized at four features with 0.775 (95% CI, 0.759 to 0.790)

The model's performance was enhanced through iterative removal of non-discriminatory features, as depicted in Fig. 2A-C. Area under the curve (AUC) reached its highest peak of 0.808 (95% CI, 0.791 to 0.825) when using 5 features: depression, joint pain, hypothyroidism, gastroesophageal reflux disease (GERD), and asthma. Sensitivity of 0.766 (95% CI, 0.743 to 0.790), F1-score of 0.761 (95% CI, 0.744 to 0.778), and accuracy of 0.775 (95% CI, 0.759 to 0.790) were achieved with the subsequent feature set, after excluding asthma. Finally, precision reached a peak of 0.817 (95% CI, 0.795 to 0.840), with the following feature set, after excluding joint pain. For model interpretation, we focused on the model involving 4 features (depression, joint pain, hypothyroidism, and GERD), as it achieved the highest number of best performing metrics.

### Model interpretation

**Fig. 3:**
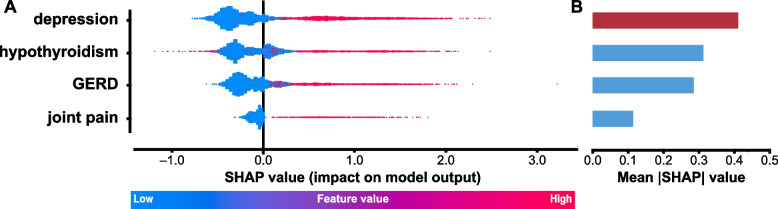
Beeswarm plot and bar graph summarizing SHapley Additive explanation (SHAP) values of the optimal model. **A** The beeswarm plot presents the SHAP values for key features—namely depression, hypothyroidism, GERD, joint pain—ranked according to their relative valence. This set of features was selected from the model with the highest performance indices. Each dot represents a predicted case, and color reflects the original value of a feature (red = feature present, blue = feature absent). For each prediction, a positive SHAP value on the x-axis indicates an increase in the model’s tendency to characterize the patient as having stiff person syndrome (SPS) and vice versa. Notably, the absence of any of these features tends to steer the classifier's prediction towards anti-GAD65 controls. **B** The bar graph demonstrates the overall absolute impact of the selected features in the models’ decision-making process. Depression emerges as the feature with the highest impact on the optimized model’s decision. Collectively, the plots visually underscore the discriminative power of these features in SPS diagnosis

Throughout the feature selection process, the SHAP values of each feature were calculated. A positive SHAP value of a feature indicates a shift in the model’s decision to characterize an individual as having SPS. To visualize the impact of identified features (depression, joint pain, hypothyroidism, GERD) on the optimal model’s decision to characterize patient as having SPS or not, their SHAP values were plotted in a beeswarm plot (Fig. 3A). It is noteworthy that the absence of each of these four items consistently influenced the classifier's decision toward anti-GAD controls. Figure 3B shows bar graphs calculated from averaged SHAP values indicating the absolute impact of the features, regardless of their directional contribution. Depression emerged as the final remaining feature, highlighting its dominant influence in characterizing SPS.

## Discussion

We leveraged EHR and employed a data-driven feature selection approach (i.e. CSA) to identify medical history items that optimally characterize SPS. By using ML models to classify patients with SPS and anti-GAD65 controls, our methodology allowed us to determine clinical features that may be linked with the diagnosis of SPS: depression, hypothyroidism, GERD, and joint pain. This study, to the best of our knowledge, is the first attempt to identify features and disease interrelationships from otherwise underutilized health information in a primary RND.

Our findings align with results from comorbidity studies and case reports pertaining to SPS [16, 20, 21]. Depression emerged as the most characteristic feature, which corroborates the established higher risk of depression and other psychiatric comorbidities in patients with SPS compared to the general population [16]. Given the chronic nature of SPS, the persistent pain these patients endure may render them more susceptible to depression [22]. Hence, characterizing the quality and progression of depression patients with SPS experience (e.g. depressed mood from chronic pain, anhedonia, sleep difficulties, etc.) may help guide the diagnosis of SPS. Furthermore, there is growing evidence that inflammatory neurologic disorders, like multiple sclerosis, may increase the risk of affective dysregulation [23, 24]. Given the presence of depression as the strongest characterizer of SPS, studying the relationship between neuro-inflammation and depression in SPS patients may lead to more targeted interdisciplinary therapies.

The primary utility of our approach is less to develop a diagnostic algorithm for immediate use, but to generate new hypotheses for RNDs even when the pathological mechanisms are not yet fully characterized. This is exemplified by our identification of hypothyroidism as one of the final discriminative features of SPS. This not only supports the suspected autoimmune pathogenesis of SPS [12], but also prompts further inquiry to examine a potential genetic association between SPS, type I diabetes mellitus, and autoimmune thyroid disorders (e.g. Hashimoto thyroiditis) through a common HLA subtype. Similarly, our identification of GERD, which has been reported to be significantly associated with depression [21], as one of the final characteristic features of SPS highlights the utility of our framework in uncovering poorly understood extra-axial dysautonomic manifestation of SPS involving the gastrointestinal tract [25, 26]. As the recognized role of autoantibodies in multiple gastrointestinal dysmotility disorders, including anti-GAD65 in diseases implicated in GERD [26, 27], continues to increase, further research may shed light on a potential pathophysiology that is common in both central and autonomic nervous system dysfunctions of SPS.

RNDs are one of the most frequently studied groups of rare diseases [28]. However, patients with a broad spectrum of RNDs continue to experience delays (approximately 4 years) in accurate diagnosis following symptom onset, which is particularly concerning for illnesses where early intervention improves prognosis and quality of life [3]. ML is transforming medicine by unearthing previously unrecognized disease associations and enriching physicians’ fund of knowledge [7]. And together with artificial intelligence, such innovation synergistically complement physicians’ diagnostic precision and accuracy whilst optimizing their anticipation of relevant comorbidities. Our feature identification strategy via CSA represents a scalable application of ML in maximizing the utility of EHR to inform future research for RNDs, thereby circumventing the constraints of statistical underpowering. Together with hypothesis-driven investigations, the integration of ML can accelerate translational research and accommodate personalized care for patients with RNDs.

One limitation of our study is the high features-to-instances ratio intrinsic to the epidemiology of SPS, which amplified the complexity of feature selection. As a proof-of-principle study, we sought to mitigate this constraint, which also affects traditional statistical models, as well as the absence of an external validation cohort by implementing a repeated-stratified validation scheme within our available cohort. Some forms of SPS may be linked with other immune processes (e.g., myasthenia gravis or progressive encephalomyelitis with rigidity and myoclonus) or represent a paraneoplastic process, such as in the context of positive anti-amphiphysin antibodies [29–31]. Our sample size limitation restricts our ability to identify such associations. Future work should aim to optimize this features-to-instances ratio and externally validate the ML models by applying the identified feature set on more comprehensive data formed by multiple institutions and networks. Another shortcoming of our study pertains to the heterogeneity inherent to EHR. We addressed this by categorizing and, in some instances, combining some symptoms and diagnoses. For example, due to the paucity of cases under each category, depressive symptoms and major depressive disorder were consolidated into a single category: “depression.” Future interdisciplinary studies that incorporate multimodal data including neuropsychiatric questionnaires and electromyography findings will provide a more complete understanding of the individual subjects.

## Conclusions

Our ML-based feature selection approach enabled the processing of underutilized EHR and identified pertinent medical items that warrant further research to fully characterize SPS: depression, hypothyroidism, and GERD may reflect comorbid conditions of SPS linked by inflammation, genetics, and the autonomic nervous system. This data-driven approach can complement more targeted investigations to explore RNDs and refine their diagnostic criteria.

## Data Availability

The data that support the results of this study are available from the corresponding author upon reasonable request.

## Abbreviations

RND: Rare neurologic disease
ML: Machine learning
EHR: Electronic health records
SPS: Stiff person syndrome
GAD-65: Autoantibodies against glutamic acid decarboxylase 65
CSA: Contribution selection algorithm
SHAP: SHapley Additive exPlanation
SVM: Support vector machine
AUC: Area under the curve
GERD: Gastroesophageal reflux disease
